# Pacemaker lead perforations: a five-year study from a high-volume center in India

**DOI:** 10.1186/s43044-025-00694-4

**Published:** 2025-10-10

**Authors:** Mohd Iqbal Dar, Imran Hafeez, Sheikh Mohamad Tahir, Jan Mohd Sheikh, Farooq A. Ganie, Syed Bilal, Ajaz A. Lone, Hilal A. Rather

**Affiliations:** Sher-i-Kashmir Institute of Medical Sciences, Srinagar, India

**Keywords:** Pacemaker lead perforation, RV perforation, CIED lead perforation, Pericardial effusion, Pericardial tamponade

## Abstract

**Background:**

Device therapy for various cardiac rhythm disturbances has seen a tremendous increase in recent times, and so have the various complications associated with this therapy. Pacemaker lead perforation is one of the most feared complications associated with these device implantations. This prospective observational study was conducted to evaluate the clinical features, diagnosis, and outcome of pacemaker lead perforation in our setting.

**Results:**

A total of 5493 patients were included in the study. It included 3438 temporary pacemaker (TPM) lead placements and 2055 patients who had undergone CIED implantation. The comorbidities of the study population include hypertension in 3582(65.21%), Diabetes in 2089(38%), dyslipidemia in 2293(41.74%) and hypothyroidism in 1527(42.6%). The indication of TPM lead implantation include complete heart block (CHB) in 1323(38.48%), TPM during CIED implantation in Sick sinus syndrome (SSS)/trifascicular block and high-grade AV block 766(22.28%), permanent pacemaker generator replacement 330(9.95%), EP study250(7.27%), drug induced heart block 13(0.38%). Indications of CIED implantation include CHB in 1103(53.67%), SSS in 221(10.75%), DCM in 132(6.42%) and ICD in 38(1.85%). There were 23 lead-induced RV perforations, with an incidence of 0.42%. There were 18(78.2%) perforations due to TPM Lead and 5(21.8%) due to CIED leads. Bradycardia was seen in 18(78.3%), hypotension in 8(34.8%), capture loss in 14(60.87%), pain abdomen in 4(17.4%). Pericardial effusion developed in 19(82.6%), tamponade needing pericardiocentesis was seen in 8(34.78%). Surgical intervention was required in 1(4.34%) case. With one death mortality in the study was 4.34%.

**Conclusion:**

Careful monitoring and nonsurgical management of lead perforation has favourable outcomes.

## Background

Cardiac rhythm disturbances requiring cardiac implantable electronic device (CIED) implantations, have shown a steady global increase. This is mainly contributed by an ageing population, increased survival of patients with various comorbidities and increasing recognition and expanding indication of various CIEDs [1]. In majority of cases, patients needing CIED implantations report to the hospital with severe unstable cardiac rhythm and more often than not need placement of a temporary pacemaker lead in the right ventricle (RV) as a bridge therapy till the final permanent device is implanted or the pacing support is no longer needed [2]. Temporary RV pacing is also often needed to provide pacing support during various structural and revascularization therapies in acute coronary syndrome, like inferior wall myocardial infarction with bradyarrhythmia or heart block.

The presence of pacing leads in the right ventricle, temporary   or permanent, is associated with various complications. Among these complications, forward displacement of leads leading to perforation of the right ventricular apex, interventricular septum or right ventricular outflow tract, is one of the most feared complications with a high mortality and morbidity [3]. Right ventricular apex remains the most common site of lead perforation, due to the fact that it is the commonest site of lead implantation. Additionally, RV apex is the thinnest part of the right ventricle and is prone to perforation. The risk of temporary pacemaker (TPM) lead perforation increases with the increase in time patients remain on TPM. CIED leads also have a potential to perforate. CIED lead perforation is classified depending upon the time of occurrence of perforation. Perforations occurring within 24 h of implant are classified as acute lead perforations, perforations occurring from 24 h to 30 days of implant as subacute and those occurring after 30 days are classified as chronic lead perforations [3, 4]. The current study was conceived to examine the clinical profile, associated signs and symptoms, incidence and outcomes of lead perforation in our setting.

## Material and methods

This was a prospective, observational, cross-sectional study conducted at our center from July 2019 to June 2024. At our center, on average, 400–500 CIED implantation procedures and 600–700 TPM procedures are performed annually. The clinical data of the patients in terms of demographic profile, age, sex, underlying clinical indication of CIED or Temporary pacemaker placement, associated co-morbidities, development of cardiac lead perforation, associated symptoms of lead perforation, development of pericardial effusion and clinical outcomes of lead perforation were noted. Ethical approval was obtained from the institutional ethics committee (SKIMS-IEC). All the patients who developed lead perforation during the study period were included in the study.

In all cases requiring a temporary pacing support, a 6 French bipolar transvenous pacing lead by St Jude Medical (St Jude Medical, DeVeaue place, Minnetonka, MN, USA) was used. The lead was placed via the femoral or transjugular route under fluoroscopic guidance. The jugular vein was the preferred route of TPM lead placement. All temporary lead tips were placed at the RV apex to ensure stability of pacing lead position and function. The permanent pacing leads used in CIED implants were active fixation, bipolar leads from Medtronic (Medtronic, 710 Medtronic Parkway, Minneapolis, MN, USA), Abbott (Abbott Medical, 15,900 Valley View Court, Sylmar, CA, USA) or Biotronic (Biotronic SE&KG, Woermannkehre, Berlin, Germany). The leads were implanted at the RV apex under fluoroscopic guidance.

**Fig. 1:**
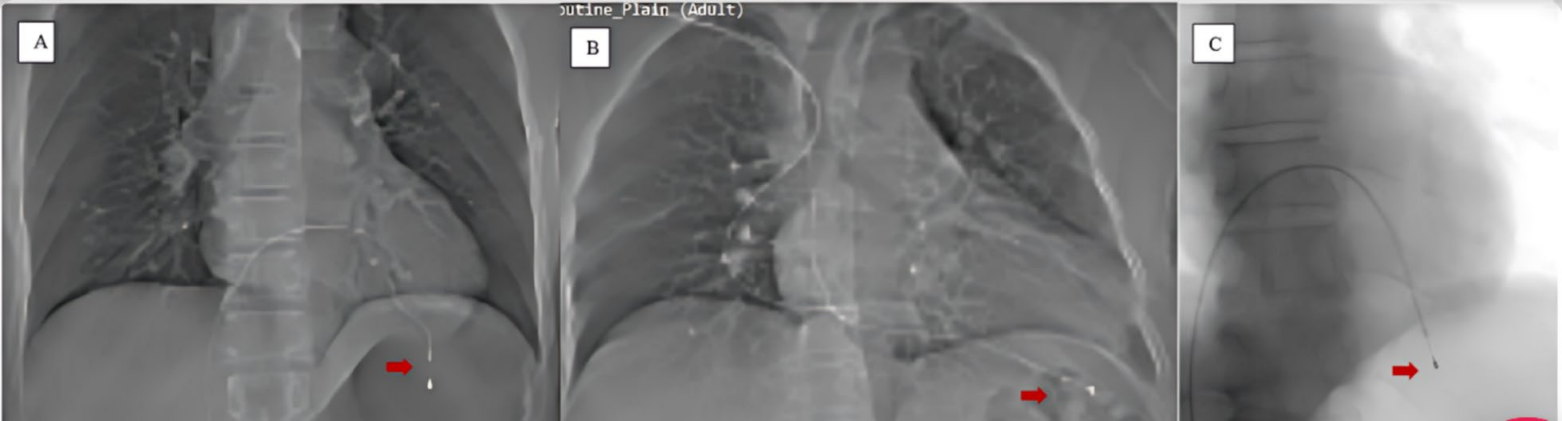
chest X-ray (**A**, **B**) and fluoroscopy (**C**) images showing temporary lead perforation (red arrows) in three different patients

**Fig. 2:**
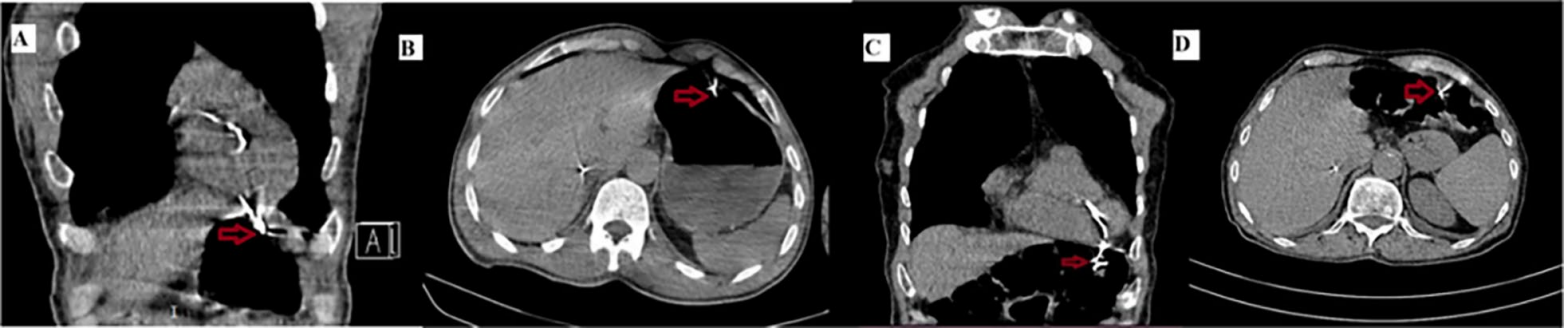
Coronal (**A**) and transverse section (**B**) CT images of one patient showing temporary lead perforating RV, diaphragm and fundus of stomach(arrows). Coronal (**C**) and transverse section (**D**) CT images of another patient showing temporary lead perforating RV, diaphragm and transverse colon (arrows)

In case of suspected lead perforation, the diagnosis was confirmed by imaging methods like echocardiography, fluoroscopy/chest X-ray (Fig. 1) and often by non-contrast CT scan of lower chest and upper abdomen in cases where the lead perforation was not clear or doubtful on fluoroscopy (Fig. 2). CT scan was also used to assess whether any other organ in the abdomen was perforated in case of suspicion.

Patients with lead perforation usually present with symptoms of bradycardia and hypoperfusion due to pacing failure, and in severe cases, the presentation can be syncope or cardiac arrest. Patients usually do not develop pericardial effusion before the temporary lead is pulled out, as the lead plugs the hole in the RV it has created. In our study, after confirmation of the diagnosis, Patients were shifted to the catheterization lab and the CVTS surgeon was informed. Blood grouping of patients was done, and 2 units were kept arranged and cross-matched.

In case of TPM lead perforation, initial management included putting in a new temporary lead in RV, connecting it to a temporary pacemaker generator and fixing the lead after attaining satisfactory parameters. The new lead was preferably placed at the mid-interventricular septum, if stable pacing parameters could be achieved. The lead causing perforation was carefully pulled out, and patients were closely monitored for the development of pericardial effusion/tamponade by echocardiography and hemodynamics. In case cardiac tamponade develops, pericardiocentesis was done. In most cases, this management would suffice. In case of continuous drainage, the patient was sent for surgical management of the RV perforation by an already awaiting surgical team.

**Fig. 3:**
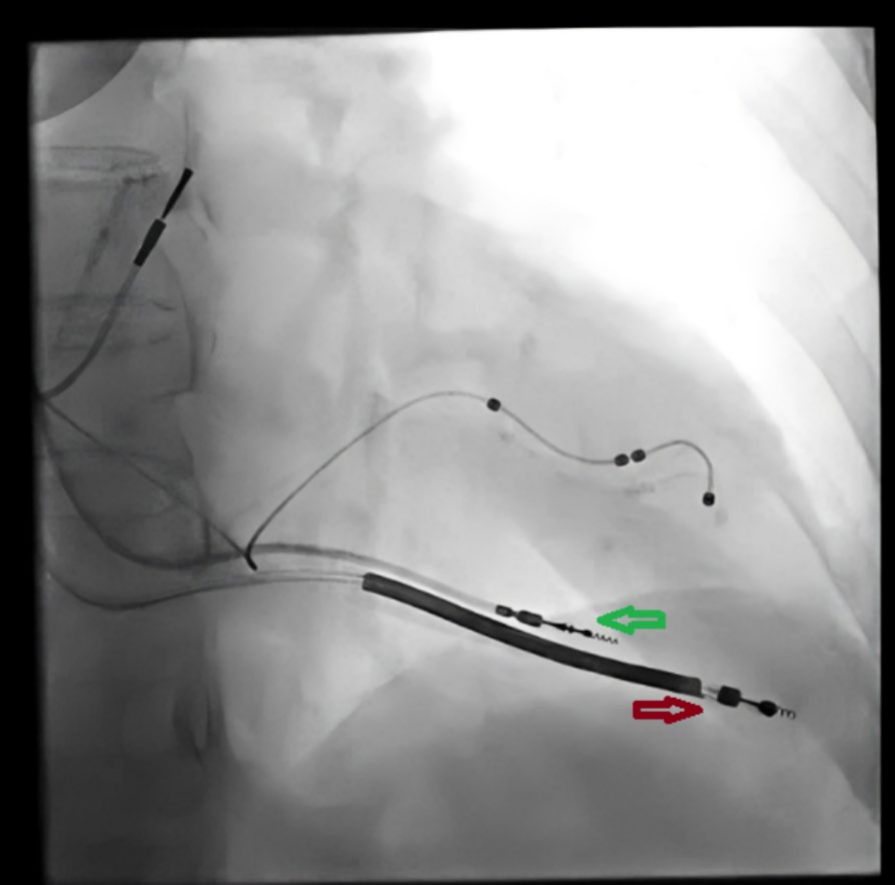
Perforation of RV by a CRT-D defibrillation lead (Red Arrow). Using LV lead for backup pacing, a new active fixation RV lead (Green arrow) is placed in RV before the perforating defibrillation lead is pulled out

**Fig. 4:**
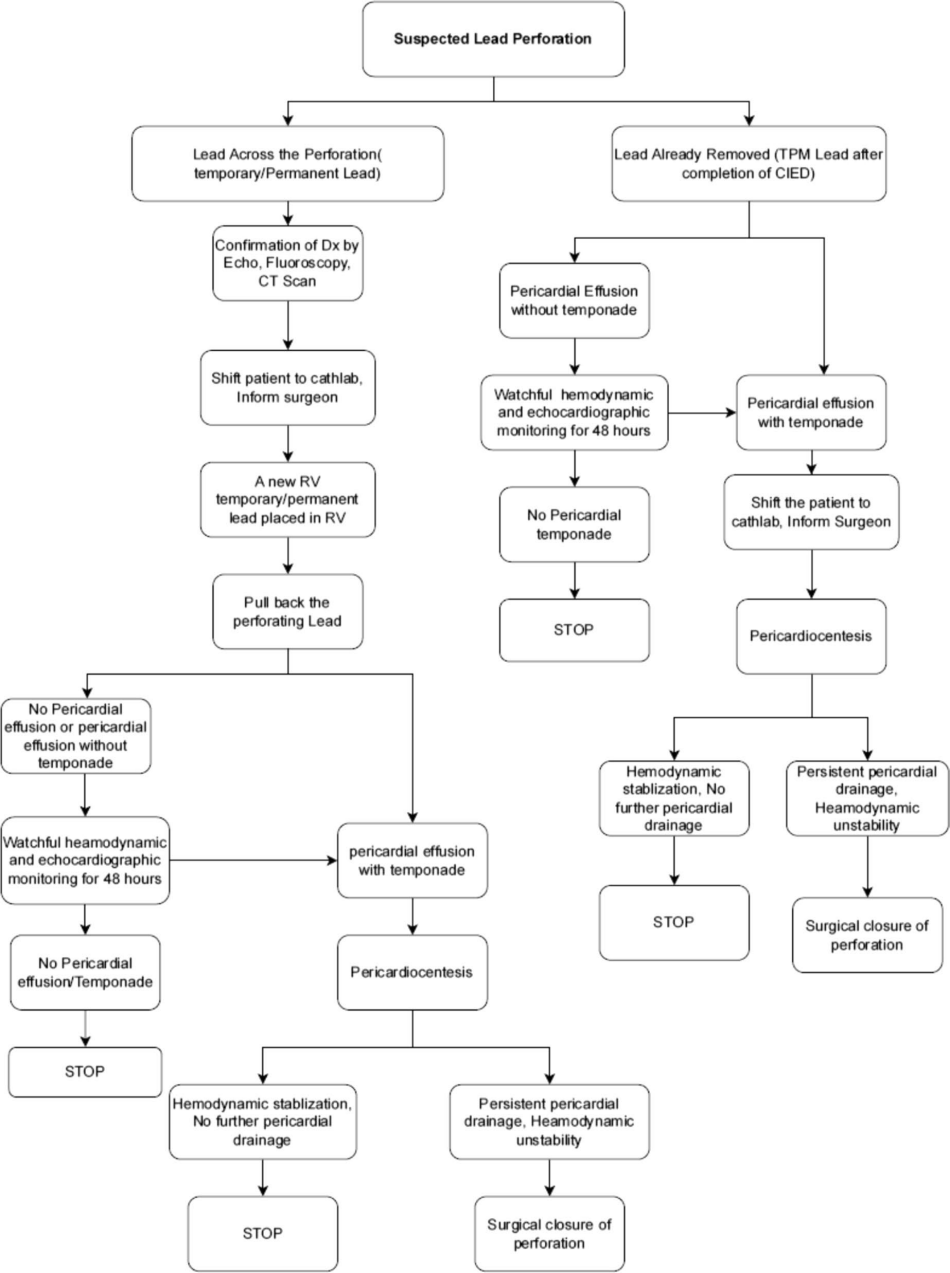
Skims protocol for pacing lead perforation management

In patients on CIEDs with permanent pacing lead perforation, the diagnosis was confirmed by echocardiography, fluoroscopy or non-contrast CT scan as needed. Keeping the surgical team informed, a TPM lead was put in RV for pacing in case the patient had an unstable cardiac rhythm before a new permanent pacing lead was put in. In patients who were on CRT, the LV lead was used for pacing backup for the procedure, sparing the need for a TPM lead for pacing (Fig. 3). The new permanent pacing lead would preferably be implanted in the RV septum, and if satisfactory pacing parameters could not be achieved, the lead was carefully placed at the proximal part of the RV apex. The lead causing RV perforation was carefully pulled out, and the patient was monitored for the development of pericardial effusion or tamponade by serial echocardiography and continuous hemodynamic monitoring. In case of development of cardiac tamponade, pericardiocentesis was done. If the drainage continued, the patient was sent for surgical intervention according to the protocol for lead perforation management of our center (Fig. 4).

### Data analysis

Continuous variables were summarized with mean ± standard deviation (SD) or median with interquartile range (IQR). Categorical variables were reported as counts and percentages. Significant differences were defined by a 2-tailed *p* < 0.05 from T-test for continuous data and Chi-square test for categorical variables. All analyses were performed using IBM SPSS version 23.

## Results

A total of 5493 patients were included in the study. It included 3438 patients who had undergone temporary pacemaker (TPM) implantation and 2055 patients who had undergone CIED implantation during the study period. The demographic profile of the study population and their associated comorbidities are given in Table 1.

**Table 1:** Demographic profile and associated comorbidities of the study population

			N (%)
TPM	Male		2255 (65.6%)
	Female		1183 (34.4%)
	Total		3438 (100)
	Mean age males		63.9 ± 11.35 years
	Mean age females		62 ± 11.9 years
CIED	Male		1330(64.7%)
	Female		725(35.3%)
	Total		2055 (100)
	Mean age males		64.5 ± 10.7
	Mean age females		61.8 ± 11.2 years
CO-MORBITIES	Hypertension	Males	2462 (68.7)
		Females	1120 (58.7)
		Total	3582 (65.21)
	T2DM	Males	1347 (37.6)
		Females	742 (38.9)
		Total	2089 (38.0)
	Hypothyroidism	Males	179 (05.0)
		Females	287 (08.0)
		Total	466 (8.5)
	Dyslipidemia	Males	1527 (42.6)
		Females	766 (40.2)
		Total	2293 (41.74)

*TPM* Temporary pacemaker, *CIED* Cardiac Implantable Electronic Devices, *T2DM* Type 2 diabetes mellitus

The indications for TPM and CIEDs in the study population are given in Table 2.

**Table 2:** Indications of TPM/CIED implantation in the study population

		N (%)
Indications for TPM implantation	Complete Heart Block	1323 (38.48)
	TPM during PPM implantation in Trifascicular block, SSS, high-grade AV block	766 (22.28)
	Left bundle branch area pacing	28 (0.83)
	Permanent Pacemaker Generator Replacement	330 (9.59)
	Hyperkalaemic heart Block	88 (2.56)
	Drug-induced (beta-blocker/calcium channel blocker) heart block	13(0.38)
	EP study	250 (7.27)
	LBBB/ asymptomatic bi or trifascicular block patients undergoing non-cardiac surgery	210 (6.10)
	TPM for rhythm support during coronary intervention	430 (12.51)
	Total	3438 (100)
Indications for CIED implantation	Complete heart block	1103 (53.67)
	Sick sinus syndrome	221 (10.75)
	Atrial fibrillation with heart block	25 (1.2)
	Mobitz type 2 heart block	216 (10.51)
	Symptomatic LBBB	143 (7.0)
	Symptomatic bifascicular/Trifascicular block	166 (8.10)
	Vasovagal syncope with cardioinhibitory response	11 (0.5)
	DCM (CRT-P/D)	132 (6.42)
	ICD (HCM/ARVC)	38 (1.85)
	Total	2055(100)

*PPM* Permanent pacemaker, *SSS* sick sinus syndrome, *EP* study- Electrophysiological study, *LBBB* left bundle branch block, *DCM* Dilated cardiomyopathy, *CRT-P/D* cardiac resynchronization therapy pacing/ defibrillation, *CIED* Cardiac Implantable Electronic Devices, *ICD* intracardiac cardioverter defibrillator

A total of 23 patients developed lead perforation out of 5493 study patients during the course of the study. It accounts for an overall incidence of 0.42%. The mean age of the patients developing lead perforation was 61.17 ± 9.87 years. There were 15 (65.2%) males and 8 (34.8%) females in the perforation group. Incidence in males was 0.42% and in females it was 0.42%. Overall, it included 18 (0.33%) lead perforations due to temporary leads (TPM) and 5(0.09%) lead perforations due to permanent leads of CIEDs. Among the permanent CIED lead perforations, 3 were caused by active fixation RV pacing leads and 2 were due to active fixation defibrillation leads. Groupwise incidence of lead perforation in the TPM cohort was 18/3438 (0.52%), and the CIED cohort was 05/2055(0.24%).

Baseline echocardiography, indication of TPM and CIED implantation, type of Lead placed and route of pacemaker lead implantation in patients developing lead perforation are given in Table 3.

**Table 3:** Baseline echocardiography, indication of pacing, type of lead and route of placement of lead in the lead perforation population

			N (%)
Baseline echocardiography		Normal LV function with no PAH	9(39.13)
		Normal LV function with PAH	6(26.08)
		LV systolic dysfunction, mild PAH	4 (17.40)
		RWMA in LAD Territory	1 (4.35)
		RWMA in RCA territory	3 (13.04)
		Total	23 (100)
Indication	of TPM	CHB	10 (43.48)
		Bi or trifascicular block	2 (8.7)
		hyperkalaemia	1 (4.35)
		Non-cardiac surgery	1 (4.35)
		Coronary intervention	4 (17.39)
	of CIED	CRT for DCM	4 (17.39)
		CHB	1 (4.34)
		Total	23 (100)
Type of lead perforated		TPM	18 (78.3)
		CIED Pacing Lead	3 (13.0)
		CIED Defibrillation lead	2 (8.7)
		Total	23 (100)
Route of Lead placement		Femoral Vein	14 (60.9)
		Jugular Vein	4 (17.4)
		Axillary Vein (CIED)	5 (21.7)
		Total	23 (100)

*LV* left ventricle, *PAH* Pulmonary artery hypertension, *RWMA* Regional wall motion abnormality, *LAD* left anterior descending artery, *RCA* right coronary artery, *CHB* complete heart block, *DCM* Dilated cardiomyopathy, *CRT* cardiac resynchronization therapy, *CIED* Cardiac Implantable Electronic Devices

Signs and symptoms of patients developing lead perforation are given in Table 4.

**Table 4:** Signs and symptoms of lead perforation in the study population

		N (%)
Bradycardia		18(78.3)
Hypotension		8 (34.8)
Pacing threshold	Increased pacing threshold	6 (26.09)
	Complete capture loss	14 (60.87)
	Not applicable	03 (13.04)
Pain abdomen		4 (17.4)
Syncope/Cardiac arrest		6 (26.1)

The mean duration of patients on TPM was 36.5 ± 21.67 h, with a duration ranging from 10 to 72 h. The mean duration of CIED implantation was 5.5 ± 4.3 months, with a minimum of 15 days to a maximum of 11 months. The duration of patients on TPM, the time of recognition of lead perforation and the investigations used to diagnose lead perforations are given in Table 5.

**Table 5:** Duration on TPM/CIED before perforation, Time of recognition of lead perforation and Investigation used to confirm led perforation

Duration on	TPM	< 24 h	7 (30.4)
		24–48 h	5 (21.7)
		> 48 h	6 (26.1)
	CIED	< 1 month	1 (4.3)
		1–12 months	4 (17.4)
	Total		23 (100)
Time of recognition of lead perforation		Before TPM removal	15 (65.3)
		After TPM Removal	3 (13.0)
		Before CIED Lead Removal	5 (21.7)
		Total	23 (100)
Investigation used to diagnose lead perforation	Echocardiography	Pericardial effusion without tamponade	11(47.8)
		Pericardial effusion with tamponade	8(34.8)
		No Pericardial effusion	4 (17.4)
		Total	23 (100)
	Fluoroscopy	lead displacement diagnosed	14 (60.9)
		lead displacement not clear	6 (26.1)
		Not applicable	3 (13)
		Total	23 (100)
	CT scan	RV perforation	12 (52.17)
		Not applicable	03 (13.04)
		Not done	08 (34.79)
		Total	23 (100)

*TPM* temporary pacemaker*, CIED* Cardiac Implantable Electronic Devices, *CT scan* computer tomographic scan

Overall, in 20 (87.0%) cases a new RV lead (temporary or permanent lead, whichever was indicated), was placed in RV for maintaining pacing backup before the perforating lead was pulled out. The other three cases developed delayed pericardial effusion after completing the permanent CIED implantation and thus did not need any additional pacing backup.

Overall, in the study population, pericardial effusion developed in 19 (82.6%) cases after removal of the perforating lead and no effusion developed in 4 (17.4%) cases. Out of these cases developing pericardial effusion, 8 (34.8%) cases developed pericardial tamponade requiring pericardiocentesis, whereas the remaining 11 (47.8%) cases of pericardial effusion did not develop any further increase in the pericardial effusion and, after close hemodynamic and echocardiographic monitoring, were finally discharged. Among the patients needing pericardiocentesis, 7 (87.5%) patients out of 8 (100%) patients showed improved hemodynamics after pericardiocentesis and needed no further treatment. The detailed management of the study patient population developing lead perforation is given in Table 6.

**Table 6:** Management of Lead Perforation

				N (%)
TPM Lead perforation management (A total of 18 cases)	Retraction of the lead and continuous hemodynamic and echocardiographic monitoring after implantation of a new TPM lead			15 (83.3)
	Lead perforation recognised after TPM lead removal			03 (18.7)
	Total			18 (100)
	Pericardial effusion	Pericardial effusion without tamponade		07 (38.89)
		Pericardial effusion with tamponade needing pericardiocentesis only		07 (38.89)
		Pericardial effusion with tamponade needing pericardiocentesis and Surgical Intervention		01 (5.55)
	No pericardial effusion			03 (16.67)
	Total			18 (100)
CIED Lead perforation management (A total of 5 cases)	Retraction of the lead and continuous hemodynamic and echocardiographic monitoring after implantation of a new CIED lead			05 (100)
	Total			05(100)
	Pericardial effusion		Pericardial effusion without tamponade	04 (80)
			Pericardial effusion with tamponade needing Pericardiocentesis	00(0.0)
			Surgical intervention	00 (0.0)
	No pericardial effusion			01(20)
	Total			05 (100)

*TPM* temporary pacemaker, *CIED* Cardiac Implantable Electronic Devices

The mortality rate in this study, with one death, was 4.34%. This outcome was in an elderly patient who developed pericardial tamponade and shock immediately after the removal of the temporary pacemaker lead, after completing a PPM implant. Echocardiography confirmed pericardial tamponade. Immediate pericardiocentesis was done. Patient continued to reaccumulate blood after complete pericardial drainage. Patient was transferred for surgical closure of the rent in RV. Surgery confirmed the rent in RV, and attempts to close the rent were made, however the patient expired before completion of the procedure.

## Discussion

The current study is probably the largest study on temporary and permanent pacing lead-induced cardiac perforations in recent times. This study evaluated the incidence, patterns and outcomes of lead perforation in patients receiving temporary or permanent pacing therapy. The mean age, gender distribution and indication of TPM or CIED implantation of the study population are consistent with other published studies in this regard [5].

The mean age of patients developing lead perforations was in line with other studies and consistent with the mean ages of the implantation cohort. The gender distribution was also consistent with the implantation cohort. In contrast to other similar studies, there was no higher incidence of lead perforations in female patients. Female sex has been found to be a factor associated with higher incidences of lead perforation and tamponade due to smaller size and relatively thinner RV wall [6, 7]. The probable reasons for the lower incidence of lead perforation in females, along with other preventive measures taken for the prevention of lead perforation, have been discussed further ahead.

The predominant presentation of lead perforation in our study was bradycardia, as expected, as the majority of the patients were having bradyarrhythmia as the underlying indication for pacing lead implantation. This was in contrast to other studies, where chest pain was the predominant symptom [8]. This may be due to the fact that the study population in these studies was predominantly having chronic lead perforation due to CIED leads. The predominant mechanism for bradycardia was loss of capture. It was because, in these cases, the lead was no longer in contact with the myocardium because of perforation. The lead in these cases would perforate the pericardium, diaphragm and would usually be in the abdominal cavity. In a quarter of patients, lead perforation presented with increased pacing threshold. The lead in these cases would perforate the RV apex but not completely displace from the myocardium. In another significant group of patients, syncope and cardiac arrest were the presenting symptoms due to complete capture loss and ventricular asystole. All these patients were resuscitated successfully due to close monitoring. Abdominal pain was observed in a few cases along with other symptoms of bradycardia and capture loss. All of these patients had a perforated pacing lead in the abdominal cavity; one of these cases had lead perforating the stomach, and the other had the pacing lead perforating the transverse colon on CT scan (Fig. 2). The incidence and pattern of other lead perforation symptoms were consistent with other published literature in this regard [7–9].

The prolongation of duration on the pacing lead has been identified as a factor for causing lead perforation [6, 9]. In our study, it was found that nearly two-thirds of patients developing TPM lead perforation had been on TPM pacing for more than 24 h. Similarly, in case patients on CIED developing CIED lead perforation, majority developed this complication after 1 month of implantation of the device. In majority of cases in our study, lead perforation was recognized before TPM lead removal. It is crucial for the proper management of these patients that lead perforation is suspected early by paying attention to the signs and symptoms of lead perforation before TPM lead removal. Patients usually do not develop life-threatening cardiac tamponade before lead removal, and it gives time for proper evaluation and management of these suspected lead perforation cases [8, 9].

All patients of suspected lead perforation underwent fluoroscopy for diagnosis of lead perforation. Fluoroscopy was able to diagnose the condition in nearly two-thirds of the cases and would often demonstrate the pacing lead outside the cardiac silhouette and below the diaphragm. In one-fourth of cases, fluoroscopy was not able to identify lead perforation. This was in cases where the pacing lead was not outside the pericardial cavity. These patients would usually present with increased pacing threshold. In published literature, fluoroscopic examination is usually the first investigation used for lead perforation diagnosis [10]. Non-contrast CT scan was done in more than half of the cases of lead perforation in our study. CT was especially useful in patients where fluoroscopy was not conclusive of the diagnosis [11, 12]. CT was additionally utilised to identify any abdominal organ perforation in cases of leads extending deep into the abdominal cavity. Echocardiography was used in all patients with lead perforation due to its ease of use and bedside availability. Though not highly sensitive, it could directly identify lead perforation in some cases. Echocardiography was highly useful in detecting mild pericardial effusion initially associated with lead perforation [13]. Echocardiography was used to monitor the development of pericardial effusion/tamponade after lead removal. Echocardiography was also helpful to quantify pulmonary artery hypertension, as high PAH with associated RV hypertrophy has been seen to be a protective factor against the development of RV perforation [14].

In all cases, where the pacing lead was across the perforation, a new pacing lead was placed in the RV before removing the perforating lead. This was done to maintain cardiac pacing and to prevent potential cardiac arrest during the management of lead perforation in these cases.

The incidence rate of TPM lead perforation in our study was 0.52%, which is significantly lower than the 1.5–2.5% in other studies [15]. This likely reflects the experience of a high-volume centre. During pacemaker lead implantation, a particular focus on safe lead implantation techniques like avoiding excessive pushing of the lead, minimising the excessive slack on the lead and proper care while transporting the patient, were given particular attention as part of a pre-specified protocol [8, 16]. Additionally, in patients with an expected thinner RV apical wall, like females and patients with dilated cardiomyopathy, a shallower lead placement was preferred. Often, the patients with heart block would present with encephalopathy due to hypoxia or other metabolic disturbances and would be irritable, risking TPM lead displacement and perforation. Appropriate treatment of the underlying condition and proper sedation of the patient, wherever indicated, was given to reduce the lead displacement and perforation risk. Preferring the jugular route of TPM lead placement in patients expected to be on TPM support for a prolonged duration also helps avoid complications, as it maintains limited mobility and decreases the discomfort of total restriction of mobility in the patients.

The incidence of CIED lead perforation in this study was comparable to other studies. All the CIED lead perforations in our study were seen with active fixation leads. This finding was consistent with the findings in other studies on the subject [17, 18]. Bulky defibrillation lead perforations formed a major chunk of these perforations. Defibrillation leads have been shown to be at greater risk of causing perforation due to their heaviness, causing higher force exertion/cm^2^ area, leading to a higher incidence of perforation with these leads [6, 12].

Pericardial effusion of varying degree developed in more than eighty per cent of the cases of this study, this was slightly higher than that of nearly sixty per cent seen in other studies [8]. In all cases, large pericardial effusion was evident only after removal of the pacing lead. In patients with TPM lead-related pericardial effusion, only half of the patients further developed pericardial tamponade and required pericardial drainage. In all but one of these cases, pericardiocentesis caused stabilization of the patient and needed no further treatment. In one case, pericardiocentesis failed to stabilize the patient and needed surgical intervention. Among the patients with CIED lead perforation, the majority developed pericardial effusion after lead removal. Pericardial effusion among these cases was without the development of pericardial tamponade and needed no further treatment.

Antiplatelet, anticoagulant and steroid use have been implicated as predisposing factors for the development of lead perforation and pericardial effusion [14, 19, 20]. In our study, four patients of the TPM lead perforation group were on antiplatelet and anticoagulants. These patients were put on TPM for complete heart block due to acute coronary syndrome. These patients developed RV perforation and pericardial tamponade during the coronary intervention procedure and needed pericardiocentesis and heparin reversal for the treatment.

There is a paucity of data on mortality in cases of acute TPM lead perforation, with mortalities in published literature ranging from 30 to 50% [9, 21]. The mortality in TPM lead perforation cohort in our study was significantly lower. This may be due to better TPM lead management during implantation and transport. The expected mortality in case of CIED lead perforations, according to published literature from large multicenter studies, is around 1.4% [8]. There was no mortality in the CIED lead perforation cohort in our study. This may be due to the overall lower incidence of CIED lead perforations in this study. Additionally, constant monitoring for possible lead perforation and preferred conservative management via a prespecified protocol significantly helps in achieving better outcomes in both TPM and CIED lead perforation patients.

## Limitation of the study

The main limitation of this study was that it was a single-centre study. A larger study with an even bigger patient population may help better understand and validate the results.

## Conclusion

This study highlights the significance of having a prespecified protocol and the importance of early detection of lead perforation. This study also demonstrates the importance of conservative management of pacemaker lead-induced RV perforation. Although this is in contrast to the early invasive and surgical management suggested in the majority of literature so far, by adopting a conservative management in these cases, a favourable outcome can be achieved in a majority of cases. This can also help avoid unnecessary invasive surgical intervention with its associated risks and complications.

## Data Availability

All the data used in this study is available with the corresponding author.

## Abbreviations

TPM: Temporary pacemaker
CIED: Cardiac implantable electronic device
RV: Right ventricle
LV: Left ventricle
CT scan: Computer tomographic scan
T2DM: Type 2 diabetes
CHB: Complete heart block
DCM: Dilated cardiomyopathy
LAD: Left anterior descending artery
RCA: Right coronary artery
PAH: Pulmonary artery hypertension
RWMA: Regional wall motion abnormality
ICD: Intracardiac cardioverter defibrillator
LBBB: Left bundle branch block
SSS: Sick sinus syndrome
